# Kobuviruses carried by *Rattus norvegicus* in Guangdong, China

**DOI:** 10.1186/s12866-020-01767-x

**Published:** 2020-04-15

**Authors:** Fang-Fei You, Min-Yi Zhang, Huan He, Wen-Qiao He, Yong-Zhi Li, Qing Chen

**Affiliations:** Department of Epidemiology, School of Public Health, Guangdong Provincial Key Laboratory of Tropical Disease Research, Southern Medical University, 1838 North Road Guangzhou, Guangzhou, 510515 China

**Keywords:** *Kobuvirus*, *Rattus norvegicus*, Prevalence, Genetic characteristic

## Abstract

**Background:**

Murine kobuviruses (MuKV) are newly recognized picornaviruses first detected in murine rodents in the USA in 2011. Little information on MuKV epidemiology in murine rodents is available. Therefore, we conducted a survey of the prevalence and genomic characteristics of rat kobuvirus in Guangdong, China.

**Results:**

Fecal samples from 223 rats (*Rattus norvegicus*) were collected from Guangdong and kobuviruses were detected in 12.6% (28) of samples. Phylogenetic analysis based on partial 3D and complete VP1 sequence regions showed that rat kobuvirus obtained in this study were genetically closely related to those of rat/mouse kobuvirus reported in other geographical areas. Two near full-length rat kobuvirus genomes (MM33, GZ85) were acquired and phylogenetic analysis of these revealed that they shared very high nucleotide/amino acids identity with one another (95.4%/99.4%) and a sewage-derived sequence (86.9%/93.5% and 87.5%/93.7%, respectively). Comparison with original *Aichivirus A* strains, such human kobuvirus, revealed amino acid identity values of approximately 80%.

**Conclusion:**

Our findings indicate that rat kobuvirus have distinctive genetic characteristics from other *Aichivirus A* viruses. Additionally, rat kobuvirus may spread via sewage.

## Background

Kobuviruses are widespread worldwide and associated with gastroenteritis, respiratory infections, and other clinical symptoms in humans and animals [1]. *Kobuvirus* was first isolated from a fecal sample from a patient with acute gastroenteritis in Japan, 1989 [2].

The genus *Kobuvirus*, belongs to family *Picornaviridae* [3]; its genome includes a 5′ untranslated region (UTR); a leader sequence(L); a large open frame encoding a single polyprotein of approximately 2400–2500 amino acids, which cleaves to produce three viral structural proteins (VP0, VP1, and VP3) and seven non-structural proteins (2A–2C and 3A–3D); a 3′UTR; and a poly (A) tail [4]. The 3D segment is a conserved region, while the VP1 protein is the most variable [5]. The genus *Kobuvirus* consists of six species, including *AichivirusA* (AiVA; formerly Aichi virus), *Aichivirus B* (AiV B; formerly Bovine kobuvirus), *Aichivirus C* (AiV C; formerly Porcine kobuvirus), *Aichivirus D* (AiV D; formerly *Kagovirus 1*), *Aichivirus E* (AiV E; formerly rabbit kobuvirus), and *Aichivirus F* (AiV F; formerly bat kobuvirus) [6]. Human kobuvirus, canine kobuvirus, feline kobuvirus, and murine kobuvirus (MuKV) belong to AiV A [7].

Kobuviruses have a wide host range worldwide, including humans, dogs, cats, pigs, cattle, goats, sheep, bats, rats, and mice [1, 4, 8–10]. The mouse kobuvirus was detected in *Peromyscus crinitus* and *Peromyscus maniculatus* mice in the USA in 2011 [11]. MuKV have since been detected in fecal samples from several countries, including Vietnam [6], Hungary [5], and the USA [12]. The study conducted in Vietnam showed that kobuviruses can be present in different rat species, including *Rattus argentiventer*, *Rattus losea*, and *Rattus norvegicus* [6]. Complete rat kobuvirus sequences from *Rattus norvegicus* were reported in a study conducted in Hungary [13]; however, information about Murine kobuviruses remains scarce.

The Norway rat (*Rattus norvegicus*) is the largest commensal rodent worldwide. They always reside near human activity and are therefore a concern for public health. There are no reports describing *Kobuvirus* prevalence and genetic characteristics in *Rattus norvegicus* in China. Here, we report the presence and genetic characteristics of rat kobuvirus in *Rattus norvegicus* in Guangdong, a province of southern China.

## Results

### Prevalence of rat kobuvirus

A total of 223 *Rattus norvegicus* animals were trapped. Twenty-eight (12.6%) *Rattus norvegicus* fecal samples were positive for *Kobuvirus* (3D region, 216nucleotides (nt)). Of samples from Guangzhou city, 26/135 (19.3%) were positive, while the rate was 2/88 (2.2%) among samples from Maoming city.

### Phylogenetic analysis

**Fig. 1:**
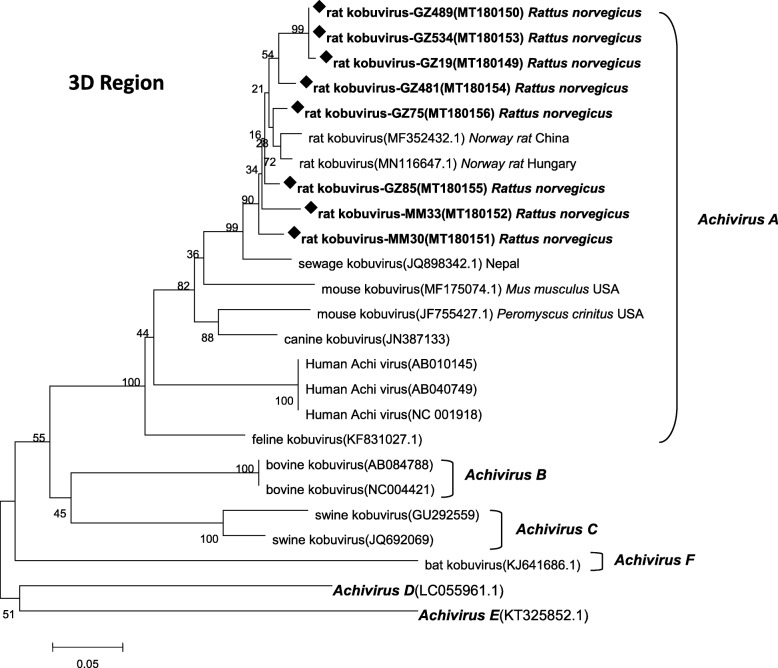
Neighbor-joining tree of *Kobuvirus* nucleotide sequences. Partial nucleotide sequences (461 bp, 7492 nt–7952 nt) were from *Aichivirus A–F* 3D genes. Bootstrap support for branches (1000 replications) is indicated. GZ481, GZ489, GZ534, GZ19, GZ75, and GZ85 (Guangzhou city), and MM30 and MM33 (Maoming city) are sequences from samples collected in this study. Rat kobuvirus sequences generated in this study are indicated by diamond symbols

Phylogenetic trees, including representative sequences detected in this study and reference sequences from Genbank, were constructed based on partial 3D sequences (461 nt) (Fig. 1). Sequences isolated from the two cities were highly conserved with one another, with nucleotide sequence identities of 92.6 to 99.8%. Further, the sequences identified in this study clustered within the *Aichivirus A* group, which also includes human kobuvirus, rat/mouse kobuvirus, canine kobuvirus, and feline kobuvirus.

**Fig. 2:**
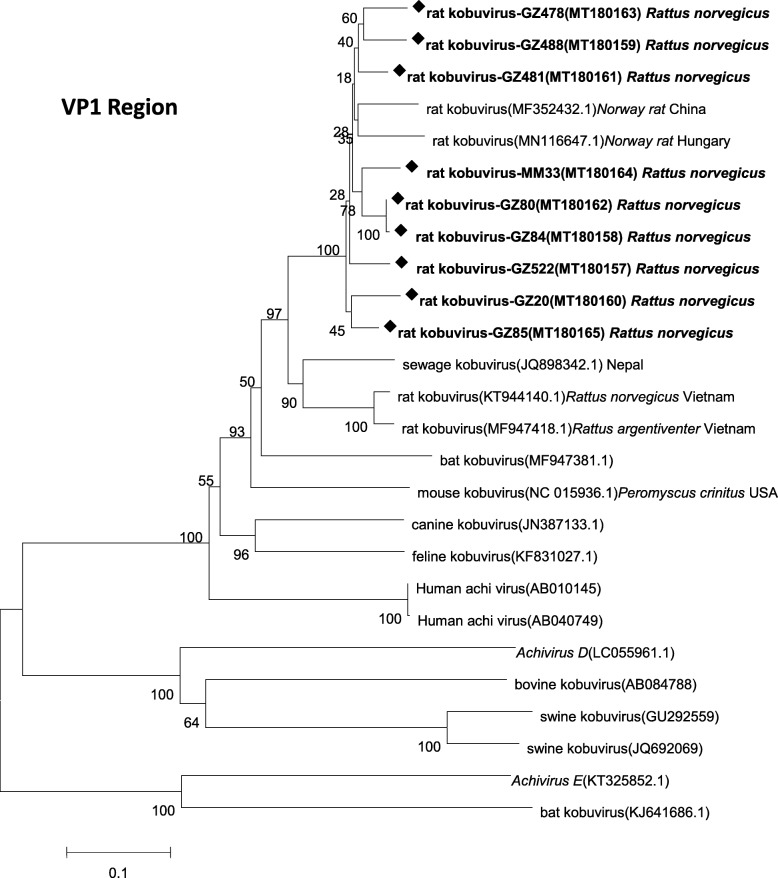
Neighbor-joining tree for *Kobuvirus* nucleotide sequences. Complete VP1 region nucleotide sequences(831 bp, 3023 nt–3853 nt) from *Aichivirus A–F* are shown above. Bootstrap support for branches (1000 replications) is indicated. GZ478, GZ481, GZ488, GZ522, GZ20, GZ80, GZ84, and GZ85 (Guangzhou city) and MM33 (Maoming city) were collected in this study. Rat kobuvirus sequences generated in this study are indicated by diamond symbols

Complete VP1 sequences were further generated from samples positive for the partial 3D gene using a one-step PCR. Sequences from several representative isolates generated in this study, along with VP1 sequences from *Kobuvirus* strains available in GenBank were used to construct a phylogenetic tree (Fig. 2). The VP1 sequences of rat kobuviruses identified in this study shared 82.1–98.6% nucleotide and 85.0–99.0% amino acid identities, as determined using DNASTAR software. Phylogenetic analysis based on VP1 nucleotide sequences showed that these sequences and two reference rat kobuvirus sequences were clustered together. Additionally, a bat kobuvirus (MF947381) [6] clustered together with those sequences rat-derived. DNASTAR software was further used to analyze the homology alignment among sequences, and the results suggested that a bat kobuvirus sequence (MF947381), derived from Vietnam, had highly similarity to the rat kobuvirus sequences detected in this study, with nucleotide (amino acids) identity values of 71.4% (75.3%) to 76.8% (84.1%); however, our sequences showed lower similarity to other bat kobuviruses reported.

### Near-complete rat kobuvirus genome sequences

**Fig. 3:**
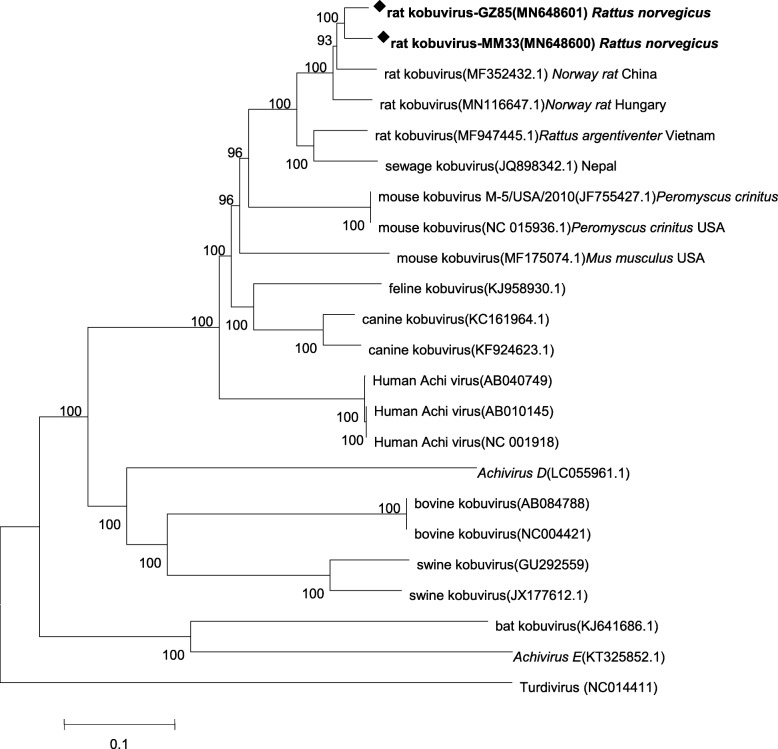
Phylogenetic relationships among near complete/complete nucleotide sequences from different *Kobuvirus* strains. Turdivirus (NC014411) was used as an outgroup for all analyses. Statistical support was provided by bootstrap analysis (1000 replicates). Rat kobuvirus sequences detected in this study are indicated by diamond symbols

Two almost full-length rat kobuvirus genomes (GZ85 and MM33; 7750 and 7734 nt, respectively) were obtained from two rat samples. A phylogenetic tree was constructed based on *Kobuvirus A*–*F* genome sequences from GenBank and these two kobuviruses (Fig. 3). Both the MM33 and GZ85 genomes showed highest nucleotide (93.44 and 93.77%) and amino acid (98.63 and 98.90%) identities to a rat kobuvirus, *Aichivirus A*, strain Wencheng-Rt386–2 (China) (MF352432.1). Further, sewage-derived kobuvirus (JQ898342.1) in Nepal and the two rat kobuviruses grouped together, with the sewage-derived sequence showing 86.9 and 87.4% nucleotide and 93.5 and 93.7% amino acid identities to the genomes detected in this study (MM33 and GZ85, respectively). Comparisons between the full sequences and L/P1/P2/P3 fragments of MM33 and reference *Kobuvirus* sequences from several other species and the environment are shown in Table 1. The MM33 genome shared a relatively high nucleotide/amino acid identity with human kobuvirus (77.4%/81.49%) and rat kobuvirus (92.8%/98.3%), respectively, while it had a low nucleotide homology with bovine (59.1%) and swine (59.3%) kobuviruses. In the P1, P2, and P3 regions, MM33 kobuvirus showed more than 70% nucleotide and 80% amino acid identity with canine, feline, rat, and human kobuviruses, which belong to the *Aichivirus A*. Further, the MM33 sequence shared > 90% amino acid sequence identity in the P1, P2, and P3 regions with the sample from untreated sewage.

**Table 1:** Putative nucleotide/amino acid identity of the rat kobuvirus (MM33) with reference *Kobuvirus* strains^a^

Gene region	Nucleotide identity (%)/amino acid identity (%)						Untreated sewage
	Human	Bovine	Swine	Feline	Canine	Rat	
L^b^	67.3/61.05	44.1/39.26	43.1/35.0	62.0/46.82	60.6/56.39	85.4/90.18	–
P1^b^	75.1/79.76	57.7/52.96	59.6/55.27	74.8/80.58	75.5/77.28	91.7/99.08	84.6/93.29
P2^b^	77.9/83.11	63.2/62.52	62.2/59.44	77.9/84.75	79.7/85.57	93.7/98.52	87.5/93.93
P3^b^	80.3/87.0	64.9/63.59	63.7/64.7	80.2/85.54	82.3/86.6	94.1/98.94	87.8/93.10
Full sequence	77.4/81.49	58.9/57.2	59.5/57.22	76.7/81.26	77.7/80.67	92.8/98.3	84.9/93.48

^a^ Reference *Kobuvirus* strains include: human (accession no. AB040749), bovine (accession no. AB084788.1), canine (accession no. JQ911763.1), rat (accession no. MN116647.1), swine (accession no.EU787450.2), feline (accession no. KJ958930.1), and untreated sewage (accession no. JQ898342.1)

^b^L: *Kobuvirus* genome leader sequence

(Human kobuvirus:745–1253 nt, Bovine kobuvirus:809–1368 nt, Swine kobuvirus:577–1160 nt, Feline kobuvirus:718–1221 nt, Canine kobuvirus:713–1225 nt, Rat kobuvirus: 737–1228 nt, Sewage kobuvirus:not mentioned)

P1: structural viral protein, including the VP0, VP1, and VP3 regions

(Human kobuvirus:1254–3791 nt, Bovine kobuvirus:1369–3939 nt, Swine kobuvirus:1161–3689 nt, Feline kobuvirus:1222–3780 nt, Canine kobuvirus:1226–3874 nt, Rat kobuvirus:1229–3853 nt, Sewage kobuvirus:181–2421 nt)

P2: nonstructural protein, including regions 2A–2C

(Human kobuvirus:3792–5699 nt, Bovine kobuvirus:3940–5841 nt, Swine kobuvirus:3690–5687 nt, Feline kobuvirus:3781–5688 nt, Canine kobuvirus:3875–5706 nt, Rat kobuvirus:3854–5686 nt, Sewage kobuvirus:2422–4329 nt)

P3: nonstructural protein, including regions 3A–3D

(Human kobuvirus:5700–8043 nt, Bovine kobuvirus:5842–8200 nt, Swine kobuvirus:5688–8043 nt, Feline kobuvirus:5689–8029 nt, Canine kobuvirus:5707–8048 nt, Rat kobuvirus:5687–8030 nt, Sewage kobuvirus:4330–6672 nt)

### Similarity plot analysis

**Fig. 4:**
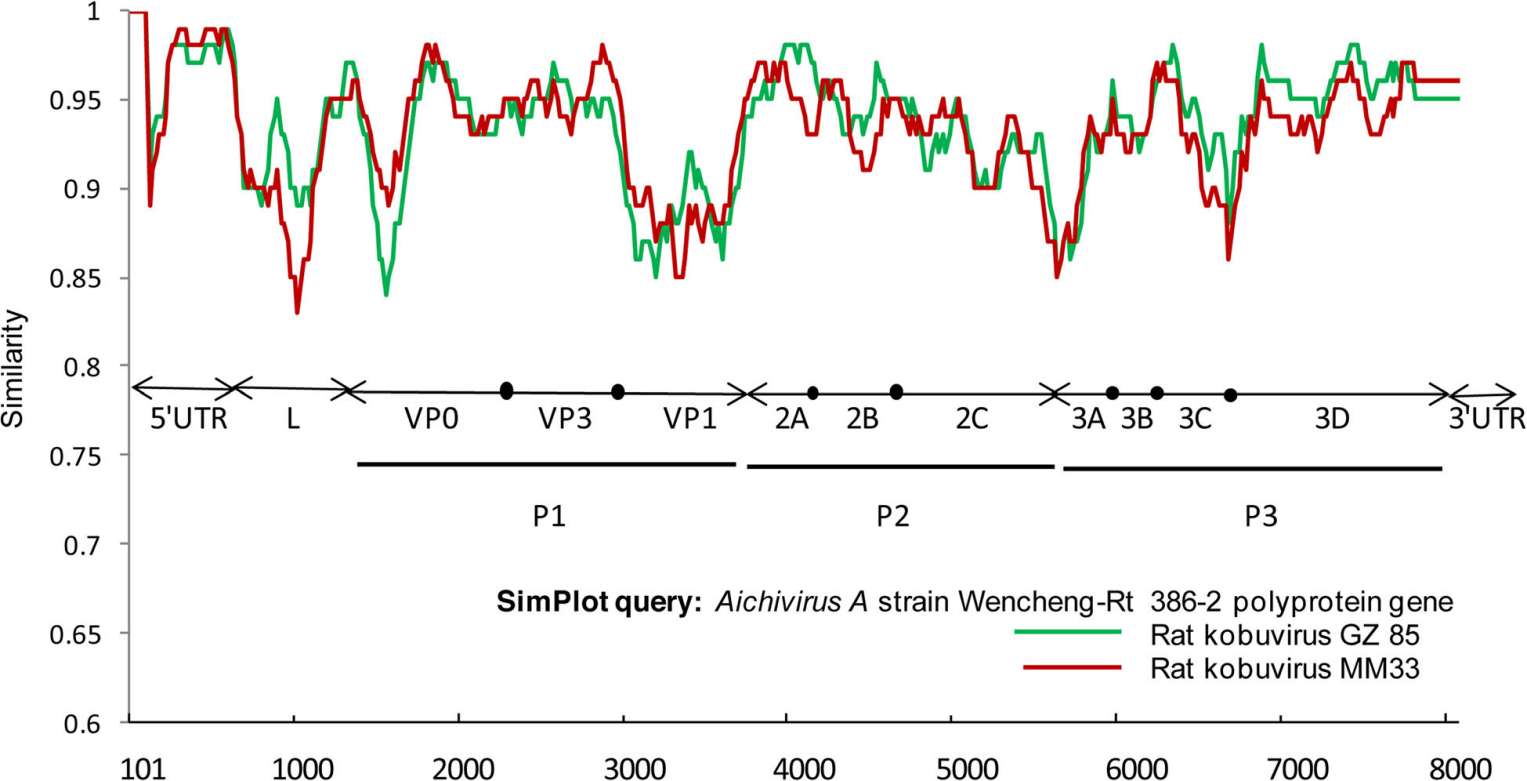
Similarity plots of the almost complete genomes of GZ85 and MM33, using MF352432.1 as the query sequence

Similarity plot analysis was conducted using SimPlot 3.5.1 to characterize the MM33 and GZ85 sequences. Standard similarity plot analysis, with the *Aichivirus A* strain Wencheng-Rt386–2 polyprotein gene (MF352432.1) as the query, showed that MM33 and GZ85 exhibited relatively high similarity to the query sequence in the 5′UTR, VP3, 2A–2C, 3B–3C, and 3D genome regions, with lower levels of similarity in the L, VP0, VP1, 3A regions, and the sequence connecting 3C and 3D (Fig. 4).

## Discussion

To our best knowledge, this is the first study to determine the prevalence and conduct genome analysis of rat kobuvirus from *Rattus norvegicus* in China. We found a mean prevalence of *Kobuvirus* in *Rattus norvegicus* of 12.6%. The animals were captured from two cities; the prevalence of *Kobuvirus* in Guangzhou was 19.3%, while that in Maoming was 2.2%. Rat kobuvirus were detected in 50% of *Rattus norvegicus* fecal samples in New York in 2014 and *Kobuvirus* are present in approximately 50% of free-living and laboratory rats (*Rattus norvegicus*) in Hungary [13, 14], while in Vietnam, the prevalence of *Kobuvirus* in rats was 17% [6]. In New York (Manhattan and Queens sites) in 2018, mouse kobuvirus was detected in 17.5% of *Mus musculus* samples. These observed variations in prevalence may be attributable to differences in rodent habitat environments. In general, our data support that *Kobuvirus* is common in rats and warrants research attention.

Phylogenetic trees based on partial 3D or VP1 regions showed that rat kobuvirus sequences cluster closely together in a clade, regardless of originating from different geographical areas. This suggests that rat kobuvirus has distinctive characteristics that differ from other *Aichivirus A* viruses. Interestingly, we found that a bat kobuvirus from Vietnam (MF947381) [6] shared high nucleotide/amino acids identity (71.4/75.3%–76.8/84.1%) with our rat-derived sequences. In addition, in another study from our laboratory, we found that part (461 nt) of *Kobuvirus* nucleotide sequences (MT180166) detected in bat feces in Guangdong showed high identity to rat kobuvirus (93.5–99.3%). These data suggest that there may be interaction between bats and rats in the environment, and offer the possibility that the viruses may have spread among different species. According to ancestral reconstruction analysis, Kobuviruses are likely to jump from rodents to bats [6]. A similar situation also occurs between bovine and swine species, with a previous finding suggesting that interspecies transmission between porcine-bovine kobuviruses may have occurred in nature [15]; however, there remains a lack of evidence that *Kobuvirus* can transmit from murine rodents to bats, or from bats to murine rodents. Hence, more evidence is required before cross-species transmission in *Kobuvirus* can be proven.

The near-complete sequences identified in this study shared more than 85% nucleotide and 90% amino acid identity to a strain from untreated sewage, similar to a report from New York [14]. These data suggest that *Kobuvirus* may transmit via water in the environment [16]. Comparison of the full sequence, MM33 with other full reference sequences revealed that MM33 showed the highest nucleotide/amino acid identity to rat kobuvirus (92.8%/98.3%), with identity values < 60% to cattle and pig sequences. Similarity plot analysis showed that MM33 and GZ85 had lower similarities at the L, VP0, VP1, and 3A loci as well as a region near the start of the 3D, indicating possible changes in these regions. These data increase knowledge of murine kobuvirus evolution.

## Conclusion

This is the first epidemiological study of rat kobuvirus in *Rattus norvegicus* in China, and demonstrates that rat kobuviruses are common in *Rattus norvegicus* feces. Rat kobuvirus have distinctive genetic characteristics that differ from other original *Aichivirus A*. Additionally, rat kobuvirus may spread via sewage. Several *Kobuvirus* nucleotide sequences detected from bat feces showed high similarity to rat kobuvirus, suggesting that interspecies transmission between murine rodents and bats may be possible. More evidence to support these findings should be sought.

## Methods

### Samples

The rats were captured close to human residences using cage traps, between January 2016 and July 2017 in two cities, Guangzhou and Maoming, about 350 km from one another in Guangdong Province. The rats were anaesthetized with diethyl ether. Species of trapped animals were determined by sequencing the cytochrome B (cytB) gene [17]. Fecal samples (approximately 0.2 g per sample) were obtained from each animal, then immersed in 700 μl phosphate-buffered saline (PBS) (0.03% homogenate), and stored at − 80 °C. After collecting stool samples, the rats were used for other studies.

### Nucleic acid extraction and RT-PCR detection

RNA and DNA were extracted from the supernatants of fecal samples using the MiniBEST Viral RNA/DNA Extraction Kit, according to the manufacturer’s instructions (TaKaRa, Kusatsu, Japan). RT-PCR was conducted using a pair of previously described primers (UNIV-kobu-F and R) to amplify a 216-nt fragment located in the conserved 3D region, encoding the RNA-dependent RNA polymerase [18]. To amplify a longer 3D region, nucleotide primer sequences were designed as follows: 3D-F (5′-CTCCGGTTGTGTGSCCACTTCC-3′; 7492 nt) and 3D-R (5′-AGCACTGCTCGCGCACTTTCAT-3′; 7952 nt), to amplify a 461 bp region. For 3D gene-positive samples, a 831 bp region of the VP1 gene was amplified by PCR using the VP1-F/VP1-R primer pair [13]. All PCR conditions were 94 °C, 3 min; 94 °C,30s, 56 °C,1.5 min, 72 °C,1 min, 40 cycles; 72 °C, 10 min. Amplicons were analyzed on 1% agarose gels followed by UV light trans-illumination.

### Full viral genome sequencing

To amplify full viral genomes, 17 pairs of primers were designed according to the GenBank reference sequence (accession number: MF352432.1) (data not shown). Lasergene SeqMan software (DNASTAR, Inc. USA, Wisconsin, Madison) was used for sequence assembly. Finally, two almost full-length rat kobuvirus genomes were obtained (GenBank accession numbers: MN648600. MN648601).

### Phylogenetic analyses

Phylogenetic analysis of Kobuviruses was performed using the Neighbor-joining method in MEGA v.6.0 (Oxford Molecular Ltd., UK). Multiple alignments were performed using the Clustal W multiple sequence alignment program in MEGA 6.0. Sequences were aligned for homology using DNASTAR software. Similarity plot analysis was conducted using SimPlot 3.5.1.

## Data Availability

All data generated or analyzed during this study are included in this published article. Access to raw data can be acquired by connecting to the corresponding author via email.

## Abbreviations

MuKV: Murine kobuviruses
AiVA/B/C/D/E/F: Aichivirus A/B/C/D/E/F
CytB: cytochrome B
